# Development and Evaluation of a Small Airway Disease Index Derived From Modeling the Late-Expiratory Flattening of the Flow-Volume Loop

**DOI:** 10.3389/fphys.2022.914972

**Published:** 2022-06-06

**Authors:** Hengji Chen, Sangeeta Joshi, Amber J. Oberle, An-Kwok Wong, David Shaz, Suman Thapamagar, Laren Tan, James D. Anholm, Paresh C. Giri, Craig Henriquez, Yuh-Chin T. Huang

**Affiliations:** ^1^ Department of Biomedical Engineering, Pratt School of Engineering, Duke University, Durham, NC, United States; ^2^ Department of Medicine, Duke University Medical Center, Durham, NC, United States; ^3^ Department of Medicine, Loma Linda University Health, Loma Linda, CA, United States

**Keywords:** small airway disease, pulmonary function test, spirometry, machine learning, CT scan

## Abstract

Excessive decrease in the flow of the late expiratory portion of a flow volume loop (FVL) or “flattening”, reflects small airway dysfunction. The assessment of the flattening is currently determined by visual inspection by the pulmonary function test (PFT) interpreters and is highly variable. In this study, we developed an objective measure to quantify the flattening. We downloaded 172 PFT reports in PDF format from the electronic medical records and digitized and extracted the expiratory portion of the FVL. We located point A (the point of the peak expiratory flow), point B (the point corresponding to 75% of the expiratory vital capacity), and point C (the end of the expiratory portion of the FVL intersecting with the *x*-axis). We did a linear fitting to the A-B segment and the B-C segment. We calculated: 1) the AB-BC angle (∠ABC), 2) BC-*x*-axis angle (∠BCX), and 3) the log ratio of the BC slope over the vertical distance between point A and *x*-axis [log (BC/A-x)]. We asked an expert pulmonologist to assess the FVLs and separated the 172 PFTs into the flattening and the non-flattening groups. We defined the cutoff value as the mean minus one standard deviation using data from the non-flattening group. ∠ABC had the best concordance rate of 80.2% with a cutoff value of 149.7°. We then asked eight pulmonologists to evaluate the flattening with and without ∠ABC in another 168 PFTs. The Fleiss’ kappa was 0.320 (lower and upper confidence intervals [CIs]: 0.293 and 0.348 respectively) without ∠ABC and increased to 0.522 (lower and upper CIs: 0.494 and 0.550) with ∠ABC. There were 147 CT scans performed within 6 months of the 172 PFTs. Twenty-six of 55 PFTs (47.3%) with ∠ABC <149.7° had CT scans showing small airway disease patterns while 44 of 92 PFTs (47.8%) with ∠ABC ≥149.7° had no CT evidence of small airway disease. We concluded that ∠ABC improved the inter-rater agreement on the presence of the late expiratory flattening in FVL. It could be a useful addition to the assessment of small airway disease in the PFT interpretation algorithm and reporting.

## Introduction

Small airways traditionally refer to the peripheral airways with an internal diameter of <2 mm (Ranga and Kleinerman, 1978). They are often considered the “quiet” zone since a disease process occurring in this region is difficult to detect clinically (Mead, 1970). This is in part because small airways that are arranged in parallel normally contribute only a small fraction of the airway resistance due to their large total cross-sectional area (Macklem and Mead, 1967). Small airway obstruction does not decrease the FEV_1_/FVC ratio, which is a measure of proximal airway function. Therefore, identifying small airway disease in pulmonary function test (PFT) represents a challenging task for the interpreters who use the current rules-based interpretation algorithm.

Changes in some parameters in PFT have been considered to infer small airway dysfunction (McNulty and Usmani, 2014). For example, increased RV/TLC indicates air trapping due to airway obstruction but is not specific to small airways (Sorkness et al., 2008). A decrease in the forced expiratory flow at 25–75% of FVC (FEF_25–75%_) is commonly cited to reflect small airway dysfunction, but it is highly variable and the value is dependent on the FVC and expiratory effort (McFadden and Linden, 1972; Boggs et al., 1982; Tager et al., 1998). The ratio of the airflow rate of 50% vital capacity to the airflow rate of 25% vital capacity (V50/V25) correlated with the difference between the R5 and R20 (R5-R20) values measured by impulse oscillometry (Suzuki et al., 2015). The forced expiratory flow at 50% FVC (FEF50%) was reduced in asymptomatic smokers who likely had small airway disease (Dosman et al., 1975). A FEF50% value less than 70% identified about 45% of patients with cough-variant asthma who had normal proximal airway function, a small airway disease phenotype (Yuan et al., 2019). Like FEF25-75%, these two measurements are also dependent on expiratory effort. Excessive decrease in the flow toward the end of the expiratory portion of a flow volume loop (FVL) or “flattening” reflects premature closure of small airways and is less effort dependent (Mead et al., 1967; Permutt and Menkes, 1979; Macdonald and Cole, 1980; Menkes et al., 1981). Studies have shown that the flow in this portion of the curve is less affected by breathing a helium-oxygen mixture, supporting the contention that this portion is from the small airways where the flow tends to be laminar and thus less density-dependent (Fox et al., 1974; Macdonald and Cole, 1980; Konstantinos Katsoulis et al., 2016).

The “flattening” is currently assessed by visual inspection of FVL by the PFT interpreters. It is subject to significant inter-rater variabilities (Hnatiuk et al., 1996). The current study sought to develop an objective index to quantify the extent of the late-expiratory flattening of the FVL. We digitized the FVLs directly from the PFT reports in PDF format from the electronic medical records and reconstructed the FVLs for the mathematical modeling. We tested the hypothesis that the availability of an objective measure to the raters would help decrease the inter-rater variability in the late-expiratory flattening of FVL. We also compared the agreement between the measure and the CT scan of the chest.

## Methods

All PFTs in this study were performed between 1/1/2018 and 10/31/2019. We downloaded the PFT reports from the Duke electronic medical record system (EPIC) that were in PDF format. PFTs that had FEV_1_/FVC >0.7 were selected. We digitized and extracted data points of the FVLs using a custom tool written in Python (version 3.7) (https://peps.python.org/pep-0537/). The study was approved by the Duke Institutional Review Board (Pro00105365).

### Flow Volume Loop Extraction and Digitization

**FIGURE 1:**
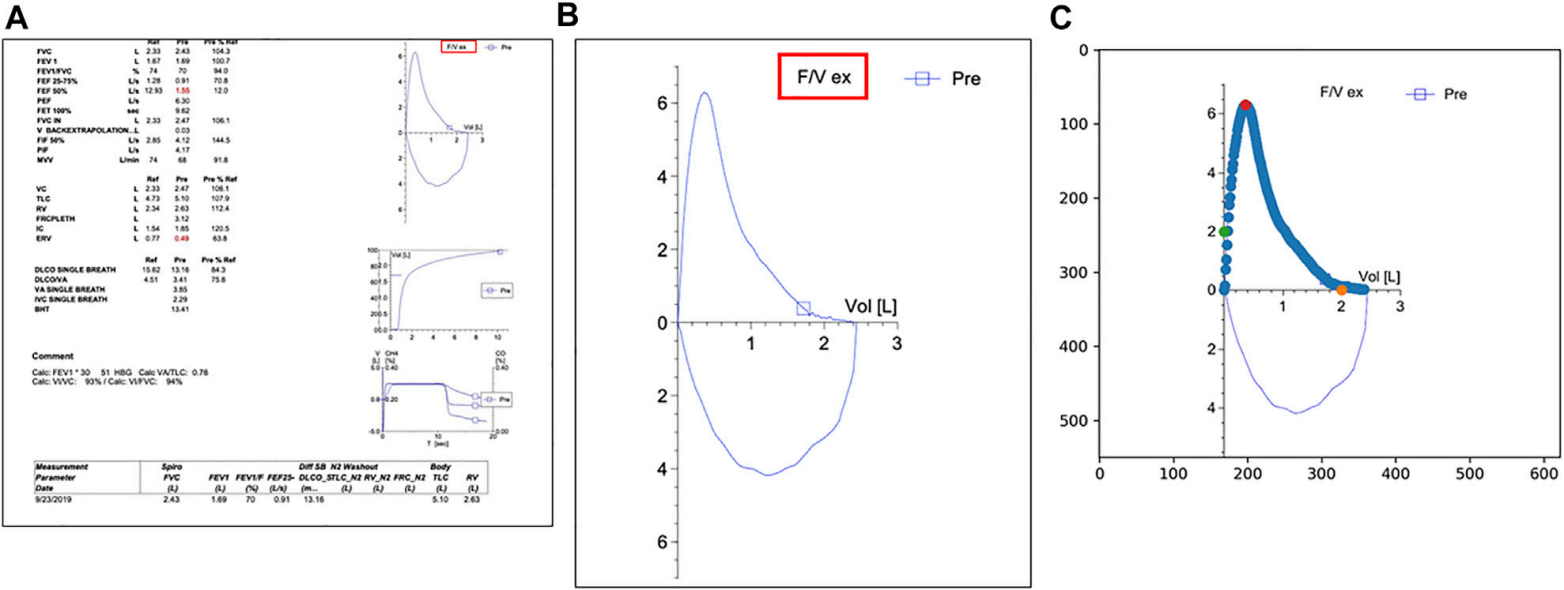
**(A)** A typical PFT report in the electronic medical record system used by Duke University Medical Center (EPIC). The label highlighted by the red box is used as an identifier to locate the flow volume loop (FVL) on the page. **(B)** The target FVL extracted by the algorithm; **(C)** The green dots and the orange dots are used for *y*-axis and *x*-axis alignment respectively. The red dot indicates the point of the peak expiratory flow.

To quantify the late expiratory flattening of the FVL, we first extracted and digitized the expiratory portion of the FVL. Since all PFT reports had the same layout with the FVL in the right upper corner as shown in Figure 1A, we identified and located the FVL by searching for the label highlighted by the red box (F/V ex) in Figures 1A,B. We calculated the image hash of the label and then conducted a search for the same hash value in the PDF report. The search was able to identify and locate the FVL in all reports. After we obtained the FVL, the *x* and *y* axes were aligned to place the pixel points along the expiratory portion of the FVL to the correct *x* and *y* scales. We first calculated the image hash of number samples such as the numeric number “2” in Figure 1B. After searching for the same image hash in the FVL, we were able to locate the number “2” on both *x*-axis and *y*-axis. Offsets were applied to the number positions so that we identified the exact tick positions for the number “2” on *x* and *y*-axis. Based on the positions of the values on the axis, we obtained the mapping coefficients describing the relationship between the pixel values and the actual value on both *x* and *y* directions. After the alignment, we picked up the data points on the curve by differentiating the pixel color from the background. These procedures allowed digitization of the expiratory portion of the FVL, which could be executed automatically.

### Quantification of Expiratory Portion of the Flow Volume Loop

**FIGURE 2:**
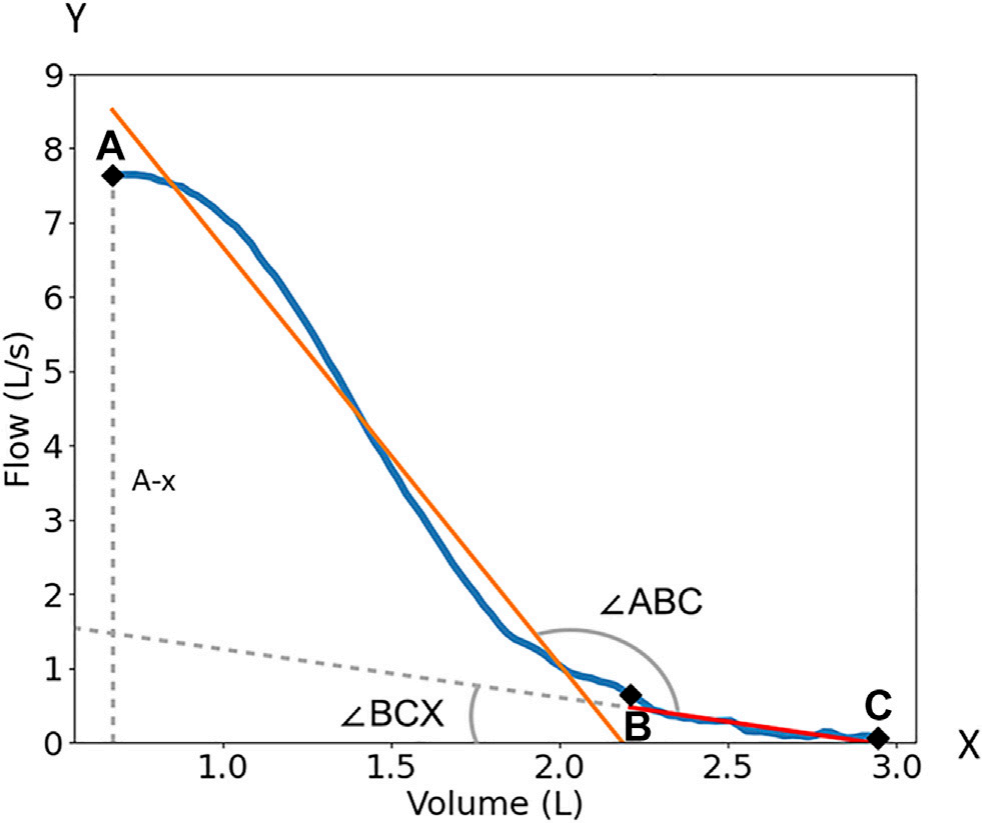
The quantification of the FVL. Point A is the peak expiratory flow of the flow volume loop; Point B is the 75% point of the maximum volume; Point C is the end of the curve. ∠ABC is the angle between the linear fitting of AB and BC. ∠BCX) is the angle between the linear fitting of BC and *x*-axis. BC/A-x is the ratio of the BC slope over peak flow or the vertical distance (dash line) between A and *x*-axis (A-x). The orange line is the linear fitting of the AB segment. The redline with dash line extension is the linear fitting of the BC segment.

After digitizing the expiratory portion of the FVL, we located the points A, B, and C on the curve as shown in Figure 2. Point A is the highest point of curve (peak expiratory flow). Point B is the point on the curve corresponding to 75% of the expiratory vital capacity, which roughly corresponds to the start of small airways (Mead et al., 1967; Wood and Bryan, 1969; Gelb and Klein, 1977). Point C is the end of the curve where the expiratory portion of the FVL intersects the *x*-axis. The A-B line segment (orange line) and the B-C line segment (green line) were used for analysis (Figure 2). We used three methods to quantify the flattening of the late expiratory portion of the FVL. These methods were designed to simulate how the clinicians visually assess the late flattening of the FVL. 1) AB-BC angle (∠ABC) is the angle between the linear fitting of AB and BC. 2) BC-*x*-axis angle (∠BCX) is the angle between the linear fitting of BC and the *x*-axis. 3) Normalized BC slope is the ratio of the BC slope over the vertical distance between A and the *x*-axis, BC/A-x. The normalization was needed because, in the PFT reports, the *x* and *y*-axes were automatically scaled to the optimal range changing the shape of the FVL. So, we include this normalized slope to mimic the automatic scaling.

### Statistical Analysis

All data are expressed as mean ± standard deviation (SD). Comparison between the two groups were performed using the unpaired student’s t-test. The concordance rate was calculated as the total number of the tests that are concordant over the total number of tests assessed. The inter-rater agreement with and without the quantification of the flattening was assessed by the Fleiss’ kappa test Fleiss and Cohen (1973). All statistical analyses were performed using Python SciPy 1.0 (Virtanen et al., 2020). A *p* < 0.05 was considered statistically significant.

## Results

### Determination of the Presence of the Late Expiratory Flattening of the Flow Volume Loop

**FIGURE 3:**
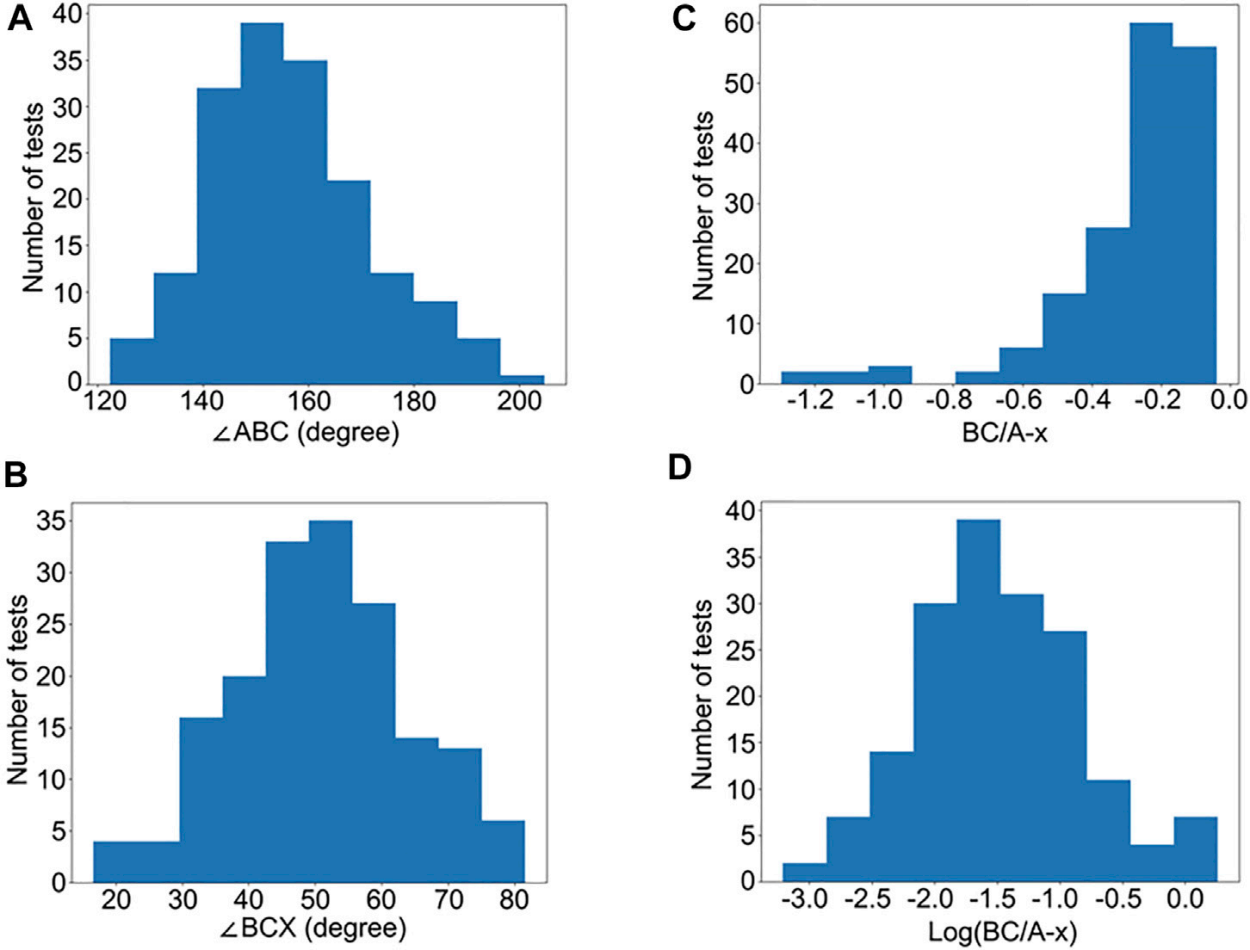
Histogram distributions of ∠ABC, ∠BCX, BC/A-x, and log (BC/A-x). The distributions of ∠ABC **(A)** and ∠BCX **(B)** are close to Gaussian while the distribution of BC/A-x **(C)** is skewed to the right. After log transformation, the distribution of log (BC/A-x) approximated Gaussian **(D)**.

We selected 172 PFTs that had FEV_1_/FVC >0.7 with adequate FVL quality. We calculated ∠ABC, ∠BCX, and BC/A-x. The distributions of ∠ABC and ∠BCX were close to Gaussian (Figures 3A,B) while the distribution of BC/A-x was skewed to the right (Figure 3C). After a log transformation, the distribution of BC/A-x approximated Gaussian (Figure 3D) and the index termed log (BC/A-x) was used for subsequent analysis.

**FIGURE 4:**
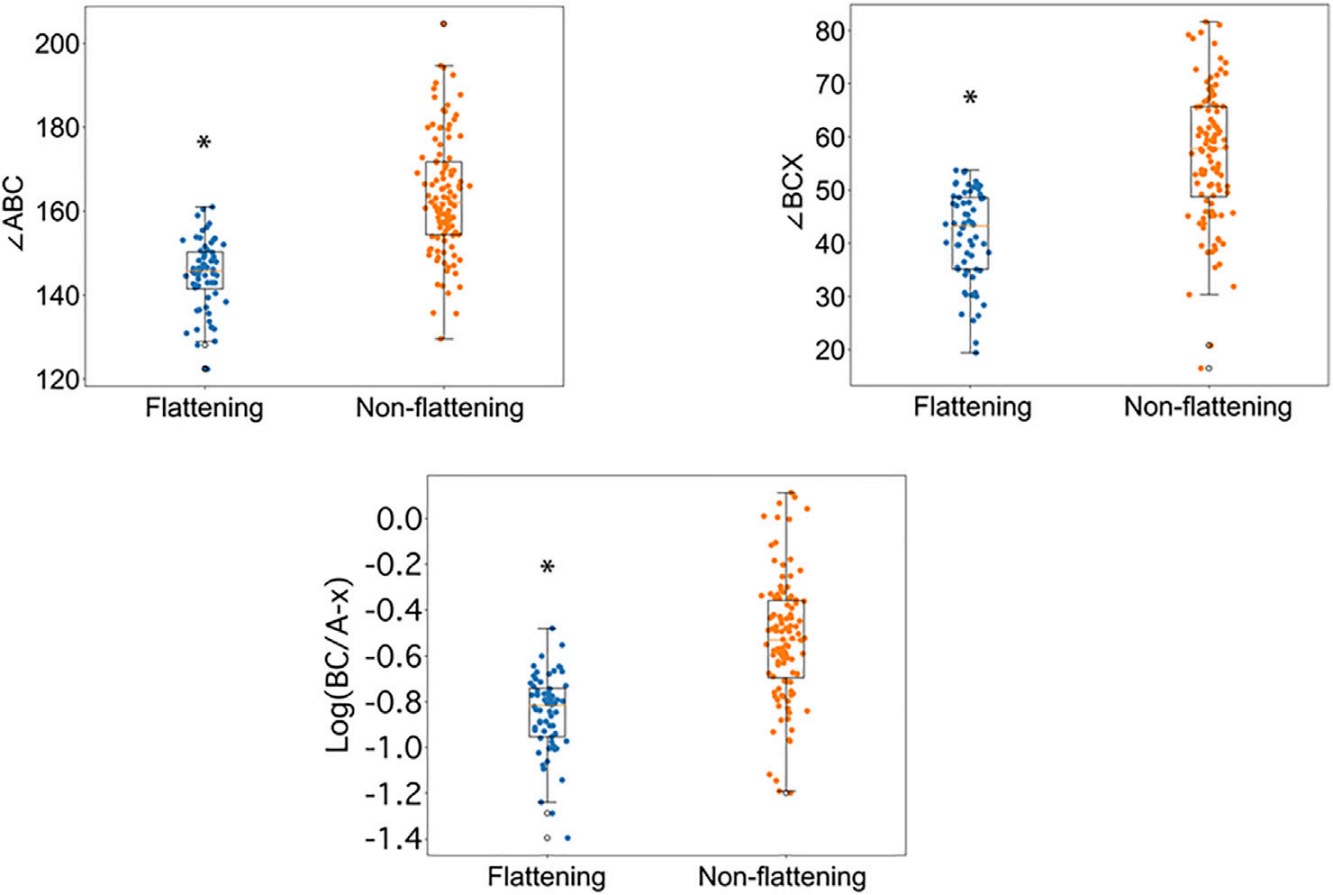
Differences in ∠ABC, ∠BCX, and log (BC/A-x) between the flattening and the non-flattening groups. The flattening and the non-flattening groups were identified by an expert pulmonologist. *: *p* < 0.0001.

Absent any additional information, an expert pulmonologist evaluated the FVLs of all 172 PFT reports to separate the tests with late expiratory flattening (*n* = 64) from those without (*n* = 108). The three metrics were computed for the flattening and the non-flattening groups. As shown in Figure 4, the mean values of ∠ABC for the flattening group and the non-flattening group were 144.8 ± 8.5° and 163.8 ± 14.1°, respectively (*p* < 0.0001). The mean values of ∠BCX for the flattening group and the non-flattening group were 41.5 ± 8.5° and 56.5 ± 12.6°, respectively (*p* < 0.0001). The mean values of log (BC/A-x) for the flattening group and the non-flattening group were −0.85 ± 0.17 and −0.52 ± 0.27, respectively (*p* < 0.0001). The concordance rates between ∠ABC, ∠BCX, and log (BC/A-x) and the expert reading were 80.2%, 74.4%, and 75.0% respectively. Because ∠ABC had the highest concordance rate, we selected it for the subsequent validation studies. We took values of ∠ABC from the non-flattening group and used the mean minus one SD as the cutoff value, which was 149.7°. When ∠ABC was less than the cutoff value, it would suggest the presence of flattening.

### Validation of ∠ABC

**FIGURE 5:**
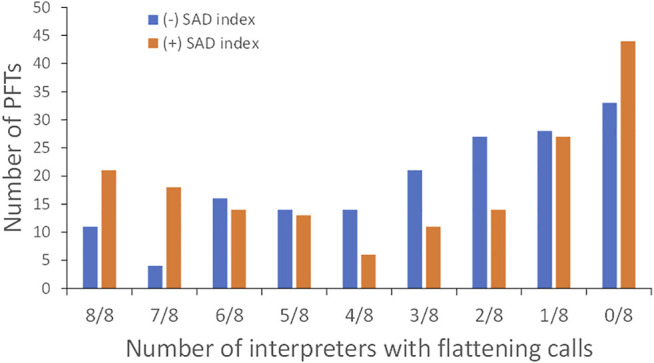
Number of pulmonary function tests (PFTs) in which the interpreters agreed to the presence of the flattening with or without ∠ABC, the small airway disease (SAD) index.

We then asked the question whether the availability of ∠ABC would decrease the inter-rater variability in assessing the flattening. Eight pulmonologists were asked to assess a different set of PFTs (*n* = 168) for the flattening with and without ∠ABC. They were first given PFTs without ∠ABC. After an interval of at least 2 weeks, they received the same set of PFTs and were asked to evaluate again with the information of ∠ABC and the instructions to use a cutoff value of <149.7° to indicate the presence of flattening. They could still disagree with the cutoff value and made their own calls. The Fleiss’ kappa without ∠ABC was 0.313 (lower and upper confidence intervals: 0.284 and 0.341 respectively). The Fleiss’ kappa with ∠ABC increased to 0.515 (lower and upper confidence intervals: 0.486 and 0.543). Figure 5 shows that the number of PFTs in which all 8 pulmonologists made the flattening calls increased from 11 to 21 when the information of ∠ABC was available to the pulmonologists. The number of PFTs in which 7 of 8 pulmonologists agreed to the presence of the flattening increased from 4 to 18. The number of PFTs in which all pulmonologists made non-flattening calls increased from 33 to 44.

### Correlation With CT Scan of the Chest

We then determined how ∠ABC correlated with small airway disease patterns in the chest CT scan. The patterns in the chest CT scan, such as mosaic attenuation or air trapping, are frequently used to imply the presence of small airway disease (Hansell, 2001; Abbott et al., 2009). We searched the electronic medical records for CT scans that were performed within 6 months of the 172 PFTs. If there were multiple CT scans associated with a PFT, the one closest to the date of the PFT was selected. We then used a text-search function in the Duke Enterprise Data Unified Content Explorer (DEDUCE) to identify reports that contained phrases, like mosaic attenuation, air trapping or small airway disease. The accuracy of the reports was further verified manually by the investigators. We identified 147 CT scans that were performed within 6 months of the PFTs. Seventy-four CT reports contained the patterns suggestive of small airway disease, and 73 did not. Of the 55 PFTs with ∠ABC <149.7°, 26 had CT scans that showed small airway disease patterns (47.3%). Of the 92 PFTs with ∠ABC ≥149.7°, 44 had no CT evidence of small airway disease (47.8%). The concordance rate between ∠ABC and CT scan was 47.6%.

## Discussion

In this study, we developed an index (∠ABC) to quantify the late expiratory flattening of the FVL using mathematical modeling. The index essentially measures the changes in flow rate during the transition from the more proximal airways to the peripheral airways. If small airway disease is present, the flow during the late expiratory phase will decrease significantly, creating a more acute angle or a smaller ∠ABC.

There were several unique steps in the development of this index. First, we digitized and extracted data directly from the PFT reports in PDF format. The PDF format is commonly used for the clinical reports in electronic medical records. Using the PDF reports avoids the challenge that may be encountered in handling raw data that are coded in various computer languages by different PFT vendors. Once digitized, the expiratory phase after the peak flow was reduced to two line segments so that different angles could be computed automatically. Finally, because there is no gold standard for small airway disease, we used an expert-supervised approach to obtain a cutoff value from the non-flattening group. This approach can recapitulate the pattern recognition capability by an expert pulmonologist who may not be readily available in many hospitals. This was an effective approach since the concordance rates between the expert pulmonologist reading and the calculated indices were high and the inclusion of one of the indices (∠ABC) on the reports improved the agreement among the interpreters.

The ability to identify small airway disease by PFT can help clinicians make early clinical decisions and potentially give a more accurate prognosis. For example, an increase in the ratio of the airflow rate of 50% vital capacity to the airflow rate of 25% vital capacity, a small airway disease marker, in the pretransplant PFT was associated with poor survival following allogeneic hematopoietic cell transplantation (Nakamae et al., 2016). A forced expiratory flow at 50% FVC (FEF50%) less than 70% identified about 45% of patients with cough variant asthma who had normal proximal airway function, suggesting probable small airway disease phenotype (Yuan et al., 2019). Our small airway disease index (∠ABC) was able to identify about 50% of the patients who had CT evidence of small airway disease. Since PFT is usually performed prior to the CT scan in outpatient visits, this can increase the pretest probability for the small airway disease and further justifies and facilitates the ordering of the CT scan.

The process described in our study for digitization, extraction and mathematic modeling can be customized for PFT reports that have different layout than ours. Specifically, the position of FVL on the report and the X-Y axes can be located by different user-defined parameters. With proper coding, the process can be automated to generate ∠ABC. The precision of the modeling requires that the PFT reports contain reasonably good quality FVLs. Therefore, clinician discretion remains an essential element in the interpretation.

In this study, we used (mean—1 SD) to define the lower cutoff value (or approximately 15th percentile). In the latest PFT interpretation guidelines, the use of the lower limit of normal (LLN) or the 5th percentile, was recommended (Culver et al., 2017). The LLN for ∠ABC was 140.8°. If the LLN was used, 17 PFTs had ∠ABC less than LLN and 6 of them (35.3%) had CT evidence of small airway disease. LLN, however, is affected by age and patient’s physical measurements. Aging is known to be associated with loss of small airway function even in subjects without airway obstruction (Martinez et al., 2017). A reference equation for ∠ABC can be generated using a larger number of PFTs and should be done in future studies.

In summary, we have developed an algorithm to digitize and extract FVL from the PFT reports in PDF format and calculated an index (∠ABC) using a mathematical modeling approach that can quantify the late expiratory flattening of the FVL. This index derived from the graphic analysis of the FVL could assist the interpreters in determining whether the late flattening was present with more confidence and thus decrease the inter-rater variability. It could be a useful addition to the assessment of small airway disease in the PFT interpretation algorithm and reporting.

## Data Availability

The data analyzed in this study is subject to the following licenses/restrictions: Ethical reasons since the database include patients’ personal health information. Requests to access these datasets should be directed to Yuh-Chin Huang, yuhchin.huang@duke.edu.
